# Supplementary material for: Dynalign II: common secondary structure prediction for RNA homologs with domain insertions
Source: Nucleic Acids Res. 2014 Nov 21;42(22):13939–48. doi: 10.1093/nar/gku1172 (PMC4267632; doi:10.1093/nar/gku1172)
Supplement: SUPPLEMENTARY DATA [file supp_gku1172_nar-02021-z-2014-File012.zip › manual/GUI/html/References.html]

RNAstructure GUI Help -- References


|  |  |  |
| --- | --- | --- |
|  | RNAstructure GUI Help References | - Contents - Index |
| **Research benefitting from RNAstructure should cite:**  J.S. Reuter and D.H. Mathews.  "RNAstructure: software for RNA secondary structure prediction and analysis."  *BMC Bioinformatics*, 11:129. (2010).   ---  The algorithms central to RNAstructure are based on the following sources. Research benefitting from these capabilities encapsulated within RNAstructure should cite the appropriate sources.  **Most current folding parameters:**  D.H. Mathews, M.D. Disney, J.L. Childs, S.J. Schroeder, M. Zuker, and D.H. Turner.  "Incorporating Chemical Modification Constraints into a Dynamic Programming Algorithm for Prediction of RNA Secondary Structure."  *Proceedings of the National Academy of Sciences USA*, 101:7287-7292. (2004).  **Previous reference to the folding algorithm, with details on thermodynamic parameter derivation:**  D.H. Mathews, J. Sabina, M. Zuker, and D.H. Turner.  "Expanded Sequence Dependence of Thermodynamic Parameters Improves Prediction of RNA Secondary Structure."  *Journal of Molecular Biology*, 288:911-940. (1999).  **Prediction of secondary structures at temperatures other than 37°C:**  Z.J. Lu, D.H. Turner, and D.H. Mathews.  "A Set of Nearest Neighbor Parameters for Predicting the Enthalpy Change of RNA Secondary Structure Formation."  *Nucleic Acids Research*, 34(17):4912-24. (2006).  **Partition function for calculating base pair probabilities:**  D.H. Mathews.  "Using an RNA Secondary Structure Partition Function to Determine Confidence in Base Pairs Predicted by Free Energy Minimization."  *RNA*, 10:1178-1190. (2004).  **Dynalign:**  D.H. Mathews.  "Predicting a Set of Minimal Free Energy Secondary Structures Common to Two Sequences."  *Bioinformatics*, 21:2246-2253. (2005).  **Extensive use of Dynalign (for extensive acceleration of the algorithm):**  A. Harmanci, G. Sharna, and D.H. Mathews.  "Efficient Pairwise RNA Structure Prediction Using Probabilistic Alignment Constraints in Dynalign."  *BMC Bioinformatics*, 8:130-150. (2007).  **Previous reference to Dynalign:**  D.H. Mathews and D.H. Turner.  "Dynalign: An Algorithm for Finding the Secondary Structure Common to Two RNA Sequences."  *Journal of Molecular Biology*, 317:191-203. (2002).  **OligoScreen:**  O.V. Mateeva, D.H. Mathews, A.D. Tsodikov, S.A. Shabalina, R.F. Gesteland, J.F. Atkins, and S.M. Freier.  "Thermodynamic Criteria for High Hit Rate Antisense Oligonucleotide Design."  *Nucleic Acids Research*, 31:4989-4994. (2003).  **OligoWalk:**  D.H. Mathews, M.E. Burkard, S.M. Freier, J.R. Wyatt, and D.H. Turner.  "Predicting Oligonucleotide Affinity to Nucleic Acid Targets."  *RNA*, 5:1458-1469. (1999).  **DNA/RNA duplex parameters for DNA oligomers:**  N. Sugimoto, S. Nakano, A. Katoh, H. Nakamura, T. Ohmichi, M. Yoneyama, and M. Sasaki.  "Thermodynamic Parameters to Predict the Stability of RNA/DNA Hybrid Duplexes."  *Biochemistry*, 34:11211-11216. (1995).  **Prediction of all suboptimal secondary structures:**  S. Dian, D.H. Mathews, and D.H. Turner.  "Interpreting Oligonucleotide Microarray Data to Determine RNA Secondary Structure. Application to the 3' End of Bombyx mori R2 DNA."  *Biochemistry*, 45:9819-9832. (2006).  **Methods for predicting suboptimal secondary structures:**  D.H. Mathews.  "Revolutions in RNA Secondary Structure Prediction."  *Journal of Molecular Biology*, 359:526-532. (2006).  **Original reference to generating all suboptimal secondary structures for a sequence:**  S. Wuchty, W. Fontant, I,L. Hofacker, and P. Schuster.  "Complete Suboptimal Folding of RNA and the Stability of Secondary Structures."  *Biopolymers*, 49:145-165. (1999).  **Stochastic sampling:**  Y. Ding and C.E. Lawrence.  "A Statistical Sampling Algorithm for RNA Secondary Structure Prediction."  *Nucleic Acids Research*, 31:7280-7301. (2003).  **Using SHAPE data to constrain structure prediction:**  K.E. Deigan, T.W. Li, D.H. Mathews, and K.M. Weeks.  "Accurate SHAPE-directed RNA Structure Determination."  *Proceedings of the National Academy of Sciences USA*, 106: 97-102. (2009).  **Photocleavage of uracil in the presence of FMN:**  P. Burgstaller, T. Hermann, C. Huber, E. Westhof, and M. Famulok.  "Isoalloxazine derivatives promote photocleavage of natural RNAs at G.U base pairs embedded within helices."  *Nucleic Acids Ressearch*, 25:4018-4027. (1997).  **ProbKnot pseudoknot prediction algorithm:**  S. Bellaousov and D. H. Mathews.  "ProbKnot: fast prediction of RNA secondary structure including pseudoknots."  *RNA*, 16:1870-1880. (2010).  **Multilign:**  Z. Xu and D. H. Mathews.  "Multilign: An algorithm to predict secondary structures conserved in multiple RNA sequences."  *Bioinformatics*, 27:626-632. (2011)  **TurboFold:**  A. O. Harmanci, H. Sharma,and D. H. Mathews  "TurboFold: Iterative Probabilistic Estimation of Secondary Structures for Multiple RNA Sequences."   *BMC Bioinformatics*, 12:108. (2011). | | |
| Visit The Mathews Lab RNAstructure Page for updates and latest information. | | |
